# Clinical characteristics and outcomes of patients hospitalized with measles during an outbreak in Somalia

**DOI:** 10.1016/j.ijregi.2023.05.003

**Published:** 2023-05-23

**Authors:** Mohamed Yaqub Hassan, Rahma Yusuf Haji Mohamud, Mohamed Mukhtar Kassim, Ahmed Issak Hussein, Mesut Kayse Adam, Ulaş Emre Akbulut, Ronald Olum, Jerom Okot, Felix Bongomin, Mohammed A.M. Ahmed

**Affiliations:** a Department of Paediatrics, Mogadishu Somalia Turkey Recep Tayyip Erdogan Training and Research Hospital, Mogadishu, Somalia; b Department of Education, Mogadishu Somalia Turkey Recep Tayyip Erdogan Training and Research Hospital, Mogadishu, Somalia; c Department of Obstetrics and Gynaecology, Mogadishu Somalia Turkey Recep Tayyip Erdogan Training and Research Hospital, Mogadishu, Somalia; d Department of Surgery, Mogadishu Somalia Turkey Recep Tayyip Erdogan Training and Research Hospital, Mogadishu, Somalia; e University of Health Sciences, Antalya Training and Research Hospital, Antalya, Turkey; f School of Medicine, College of Health Sciences, Makerere University, Kampala, Uganda; g Department of Medical Microbiology and Immunology, Faculty of Medicine, Gul University, Gulu, Uganda; h Department of Paediatrics, Faculty of Medicine and Surgery, Mogadishu University, Mogadishu, Somalia; i Department of Paediatric Cardiology, Uganda Heart Institute, Kampala, Uganda

**Keywords:** Measles, Somalia, Clinical characteristics and outcome, Outbreak

## Abstract

**Objectives:**

Measles is a contagious disease associated with significant morbidity and mortality. This paper describes the clinical characteristics and outcomes of patients hospitalized with measles during an outbreak in Somalia from January 2018 to December 2021.

**Methods:**

This retrospective study was conducted at Mogadishu Somalia Turkey Recep Tayyip Erdogan Training and Research Hospital. Children aged 6 months to 17 years who were hospitalized with clinical signs and symptoms of measles and its complications were recruited

**Results:**

In total, 110 participants were enrolled. The median age was 16 [interquartile range (IQR) 12–36] years, and 87 (79.1%) were male. All participants presented with fever, typical measles rash, cough and conjunctivitis, and 43 (39.1%) had received the measles vaccine. Overall, 104 (94.6%) participants were admitted with severe respiratory symptoms, and six (5.4%) were admitted due to poor feeding and/or significant dehydration. Overall, all-cause mortality was 1.8% (*n*=2). The median duration of hospitalization was longer among participants who died compared with those who survived [11 (IQR 8–14) vs 4 (IQR 2–6) days; *P*=0.046]. Unvaccinated participants were significantly younger than vaccinated participants [36 (IQR 24–72) vs 12 (IQR 9–16) months; *P*<0.001]. There was a trend towards higher mortality [0/43 (0%) vs 2/67 (3%); *P*=0.519] and longer length of hospitalization [3 (IQR 2–6) vs 4 (IQR 3–7) days; *P*=0.056] among unvaccinated participants compared with vaccinated participants. Both the median total leukocyte count [5.7 (IQR 3.9–8.5) vs 11.6 (IQR 5.9–46.3) x 10^9^/L; *P*<0.001] and platelet count [239 (IQR 202–358) vs 308 (IQR 239–404) x 10^9^/L; *P*=0.032] were significantly higher among unvaccinated participants compared with vaccinated participants. However, the median haemoglobin concentration was significantly higher among vaccinated participants compared with unvaccinated participants [11.1 (IQR 9.9–12.3) vs 10.1 (IQR 9.1–11.2) g/dL; *P*=0.006].

**Conclusions:**

Patients with measles in Somalia have a short hospital stay, low mortality rate and low vaccination rate. Timely vaccination and the need for improved care of patients with measles, particularly vulnerable groups including children and those with undernutrition, are encouraged.

## Highlights

• This study reports the results of children hospitalized with measles in Somalia.
• Of the 110 participants, 43 (39.1%) had received the measles vaccine.
• Two (1.8%) patients died.
• The length of hospital stay was longer among unvaccinated children.

## Introduction

Measles is an extremely contagious viral disease that can have serious consequences, including death [1]. Most deaths from measles occur in children aged <5 years [1], making it one of the leading causes of childhood mortality globally. Nevertheless, a low-cost, safe vaccine is available, and persistent international efforts have been made to eradicate the illness [2]. In order to achieve this, very high vaccination coverage (>95%) must be attained and maintained for protracted periods of time. Before the measles vaccine was launched in 1963, there were an estimated 30 million deaths attributed to measles each year, causing more than 2 million deaths [3].

Although public health initiatives have increased and vaccination rates have been sustained, outbreaks of varying intensity have continued to occur in Somalia, and the country faces almost yearly reports of outbreaks. In early 2022, suspected measles cases were reported from all over the Somali region [4].

Measles virus is mainly transmitted through direct contact with airborne respiratory droplets. The virus remains active and contagious from 4 days before to 4 days after the rash erupts. The incubation period is 10–14 days [5].

Symptoms of measles include fever, maculopapular skin rash, cough, coryza, conjunctivitis, and small white papules in the buccal mucosa (Koplik's spots, which appear 1 or 2 days before the rash and are pathognomonic) [3,6].

Complications of measles include pneumonia, tracheitis, diarrhoea, otitis media, hepatitis, encephalomyelitis, encephalitis, superimposed bacterial infections, subacute sclerosing panencephalitis and death. These complications can affect most organ systems, and are most common in young infants, adults aged >20 years, pregnant women, and those who are immunocompromised or undernourished, particularly children with vitamin A deficiency [7].

Regarding other public health interventions in Somalia, vaccine administration and measles management have been affected by internal conflicts, long-lasting civil war, droughts and lack of public health awareness, in addition to low levels of access to health care [8]. Therefore, this study aimed to describe the clinical characteristics and outcomes of patients hospitalized with measles during an outbreak in Somalia,

## Methods

### Study design and setting

This retrospective study was conducted in the Paediatric Department at Mogadishu Somalia Turkey Recep Tayyip Erdogan Training and Research Hospital, a tertiary level hospital, from January 2018 to December 2021. Patients are transferred to this hospital from all over the country. The hospital has a total of 240 beds, and as well as treating patients, serves as a training hospital for doctors and other medical professionals.

### Study population

Children aged 6 months to 17 years old, hospitalized with clinical signs and symptoms of measles and its complications, were included in this study. Children aged <6 months were excluded because they are considered to have passive immunity to measles passed to them transplacentally from their mothers.

### Sample size

All participants who met the eligibility criteria and were hospitalised during the study period were included in this study. In total, 77,105 children attended the paediatric clinic during the study period, 528 children had signs and symptoms consistent with measles, and 110 children were hospitalized.

### Study procedures

All patients who met the inclusion criteria were included in this study, and their electronic medical records were obtained. Demographic date collected included age, sex, immunodeficiency, comorbidities, previous measles exposure, pregnancy, immunization status, and repeated hospital admissions for measles.

### Statistical analysis

The data were cleaned and prepared in Excel 2016 (Microsoft Corp, Redmond, WA, USA), and then imported into STATA Version 17.0 for analysis. Normality of distribution of numerical data was assessed using the Shapiro–Wilk test. Non-normally distributed variables are presented as median and interquartile range (IQR), and categorical data are presented as frequency and percentage. Chi-squared test and Fisher's exact test were used to compare and assess associations between categorical independent variables and mortality and vaccination status, and Mann-Whitney *U*-test and independent sample *t*-test were used to assess associations between numerical independent variables and dependent variables (mortality and vaccination status). A multi-variable logistic regression model was constructed to assess independent predictors of in-hospital mortality, and the results are presented as adjusted odds ratios (OR) with 95% confidence intervals (CI) and *P*-values. *P*<0.05 was considered to indicate significance.

## Results

### Baseline characteristics of study participants

Overall, 528 (0.7%) of 77,105 children who attended the paediatric clinic during the study period were suspected to have measles, and 110 (20.8%) required hospitalization. The median age of the hospitalized cases was 16 (IQR 12–36) months, and 87 (79.1%) were male. All participants presented with fever, typical measles rash, cough and conjunctivitis. Overall, 43 (39.1%) participants had received the measles vaccine. The median leukocyte count was 7.9 (normal range 4–12) cells/mm^3^, haemoglobin was 10.6 (normal >11.5) g/dL, platelet count was 284 (normal range 150–450) cells/mm^3^, C-reactive protein was 9.4 (normal range 1–10) mg/dL, alanine aminotransferase was 20 (normal <40) U/L, and aspartate aminotransferase was 64 (normal <40) U/L. One (1%) participant had underlying immunodeficiency and one (1%) had diabetes mellitus (Table 1).

**Table 1:** Factors associated with mortality among patients hospitalized with measles in Somalia

Variable	All (*n*=110) Frequency (%)	Mortality		*P*-value
		Yes (*n*=2) Frequency (%)	No (*n*=108) Frequency (%)	
Age, median (IQR), months	16 (12–36)	13.5 (11–16)	16.5 (12–36)	0.576
Sex				
Male Female	87 (79.1) 23 (20.9)	0 (0) 2 (100)	87 (80.6) 21 (19.4)	0.042
Gastroenteritis				
Vomiting Diarhea No	43 (39.1) 58 (52.7) 9 (8.2)	1 (50) 1 (50) 0 (0)	42 (38.9) 57 (52.8) 9 (8.3)	0.891
Vaccinated				
Yes No	43 (39.1) 67 (60.9)	0 (0) 2 (100)	43 (39.8) 65 (60.2)	0.519
Length of hospital stay, median (IQR), days	4 (2–6)	11 (8–14)	4 (2–6)	0.046
White cell count, x 10^9^/L	7.9 (4.9–14.2)	10.6 (9.2–11.9)	7.8 (4.9–14.9)	0.576
Haemoglobin, g/dL	10.6 (9.4–11.5)	8.4 (7.1–9.7)	10.7 (9.5–11.6)	0.107
Platelet count, x 10^9^/L	284 (222–394)	404.5 (356–453)	280.5 (222–393.5)	0.233
C-reactive protein, mg/dL	9.4 (3.1–27.9)	56.7 (22.3–91)	8.9 (3.1–27.8)	0.168
Aspartate aminotransferase, U/L	64 (51–81)	97 (87–107)	64 (51–79)	0.097
Alanine aminotransferase, U/L	20 (14–30)	35.5 (28–43)	20 (14–30)	0.182
Diabetes mellitus	1 (0.9)	0 (0)	1 (0.9)	>0.999
Immunodeficiency	1 (0.9)	0 (0)	1 (0.9)	>0.999
Indication for hospitalization				
Respiratory symptoms Poor feeding and dehydration	104 (94.6) 6 (5.4)	2 (100) 0 (0)	102 (94.4) 6 (5.6)	>0.999
Re-admission for measles	3 (2.7)	0 (0)	3 (3.8)	>0.999

IQR, interquartile range.

### Hospitalization and in-hospital mortality

The median duration of hospitalization was 4 (IQR 2–6) days. Three (2.7%) participants were re-admitted for measles. Overall, 104 (94.6%) participants were admitted due to severe respiratory symptoms, and six (5.4%) were admitted due to poor feeding and/or significant dehydration. All-cause mortality was 1.8% (*n*=2) and both deaths were female (*P*=0.042). The median duration of hospitalization was significantly higher among participants who died compared with those who survived [11 (IQR 8–14) vs 4 (IQR 2–6) days; *P*=0.046] (Table 1). On multi-variable analysis, no significant association was found between length of hospital stay and mortality (adjusted OR 1.2, 95% CI 0.98–1.55; *P*=0.068).

### Comparison of outcomes with vaccination status

Unvaccinated participants were significantly younger than vaccinated participants [median age 36 (IQR 24–72) vs 12 (IQR 9–16) months; *P*<0.001]. There was a trend towards higher mortality [0/43 (0%) vs (2/67 (3%); *P*=0.519] and longer length of hospitalization [median 3 (IQR 2–6) vs 4 (IQR 3–7) days; *P*=0.056] among unvaccinated participants compared with vaccinated participants. Both the median total leukocyte count [5.7 (IQR 3.9–8.5) vs 11.6 (IQR 5.9–46.3) x 10^9^/L; *P*<0.001] and platelet count [239 (IQR 202–358) vs 308 (IQR 239–404) x 10^9^/L; *P*=0.032) were significantly higher among unvaccinated participants compared with vaccinated participants. However, the median haemoglobin concentration was significantly higher among vaccinated participants compared with unvaccinated participants [11.1 (IQR 9.9–12.3) vs 10.1 (IQR 9.1–11.2) g/dL; *P*=0.006] (Table 2).

**Table 2:** Comparison of baseline characteristics and clinical outcomes of the study participants by vaccination status

Variable	All (*n*=110) Frequency (%)	Vaccinated		*P*-value
		Yes (*n*=43) Frequency (%)	No (*n*=67) Frequency (%)	
Age, median (IQR), months	16 (12–36)	36 (24–72)	12 (9–16)	<0.001
Sex				
Male Female	87 (79.1) 23 (20.9)	31 (72.1) 12 (17.9)	56 (83.6) 11 (16.4)	0.159
Gastroenteritis				
Vomiting Diarhea No	43 (39.1) 58 (52.7) 9 (8.2)	19 (44.2) 20 (46.5) 4 (9.3)	24 (35.8) 38 (56.7) 5 (7.5)	0.579
Mortality				
Yes No	2 (1.8) 108 (98.2)	0 (0) 43 (100)	2 (3) 65 (97)	0.519
Length of hospital stay, median (IQR), days	4 (2–6)	3 (2–6)	4 (3–7)	0.056
White cell count, x 10^9^/L	7.9 (4.9–14.2)	5.7 (3.9–8.5)	11.6 (5.9–46.3)	<0.001
Haemoglobin, g/dL	10.6 (9.4–11.5)	11.1 (9.9–12.3)	10.1 (9.1–11.2)	0.006
Platelet count, x 10^9^/L	284 (222–394)	239 (202–358)	308 (239–404)	0.032
C-reactive protein, mg/dL	9.4 (3.1–27.9)	7.7 (3.1–24.6)	11.8 (3–37.2)	0.304
Aspartate aminotransferase, U/L	64 (51–81)	64 (55–74)	64 (50–86)	0.802
Alanine aminotransferase, U/L	20 (14–30)	20 (13–30)	20 (15–30)	0.863
Diabetes mellitus	1 (0.9)	0 (0)	1 (1.5)	>0.999
Immunodeficiency	1 (0.9)	0 (0)	1 (1.5)	>0.999
Indication for hospitalization				
Respiratory symptoms Poor feeding and dehydration	104 (94.6) 6 (5.4)	42 (97.7) 1 (1.3)	62 (92.5) 5 (7.5)	0.401
Repeat of hospitalization	3 (2.7)	0 (0)	3 (4.5)	0.279

IQR, interquartile range.

## Discussion

Measles is a highly contagious viral infection that primarily affects children. It causes significant morbidity and mortality globally [9,10]. This study aimed to investigate the clinical characteristics and outcomes of 110 patients hospitalized with measles during an outbreak in Somalia. The median age of the patients was 16 months, and 87 were male. All participants presented with fever, typical measles rash, cough and conjunctivitis. The hospitalization rate was high, with almost one in every five children with measles being hospitalized. Although only 39.1% of the participants had received the measles vaccine, this study showed that the in-hospital mortality rate was low (<2%). Unvaccinated participants were significantly younger, and showed a trend towards higher mortality and longer length of hospitalization compared with vaccinated participants. It remains unknown whether the low mortality rate observed in this study was because several cases of severe measles died in the community and did not reach hospital due to difficulties with access to health care. This is an area for future study.

The in-hospital mortality rate of 1.8% found in this study of patients hospitalized with measles during an outbreak in Somalia is relatively low compared with previous studies. A systematic review and modelling analysis found that the overall pooled mean case fatality rate for measles was 2.2%, with an in-hospital mortality rate of 2.9%, ranging from 0.9% to 6.0% [11]. A study in Pakistan reported an in-hospital mortality rate of 5.1% among children hospitalized with measles [12]. In Nigeria, the in-hospital case fatality rate reported in a 10-year retrospective study in 2007 was 7.5% [13]. The finding from the present study is similar to a large study of >1800 patients with measles in Ethiopia in 2013, which reported a case fatality rate of 1.89% [14].

The in-hospital mortality rate found in this study was lower than reports from other settings. This may be due to several factors, including the increase in measles vaccination coverage [15], greater public awareness or recognition, high hospitalization rate and timely treatment, and decreasing trends in malnutrition [16] in Somalia compared with previous decades. In addition, the low mortality rate could be attributed to the fact that the study was conducted at a tertiary level hospital, where patients with severe symptoms are more likely to receive appropriate medical care. However, it is also possible that this study may have underestimated the true mortality rate due to the study design, as some patients who died may not have been included in the study due to displacement caused by conflicts and droughts. Previous studies on Somalian refugees in Kenya and Ethiopia showed a higher mortality rate compared with the rate found in the present study [17,18].

Measles is a vaccine-preventable disease and is one of the targets of the Sustainable Development Goals (Target 3b) [19]. The vaccination rate found in this study was relatively low (39.1%). This is slightly lower than estimates from the World Health Organization (WHO) and the United Nation's Children's Fund, which indicate that coverage of measles vaccine for the first and second doses is 46% and 4%, respectively [20]. These numbers are considerably lower than the current global estimates of 81% and 71% for the first and second doses, respectively, and fall short of the 95% target set by WHO [21]. Moreover, a study among children born in Norway by Somali immigrants showed that up to 87% were vaccinated against measles. However, the vaccination rate seen in this study was higher than the rates of 20.4% and 6% reported in Somali refugees in Kenya and Ethiopia [17,18]. These trends point to the importance of increasing investments into interventions aimed at increasing vaccination coverage in Somalia, including improving access to key populations, especially those facing conflicts.

The body's immune response to measles is complex, and involves both the innate and adaptive immune systems [22]. The innate immune response, which occurs immediately after exposure to the virus, includes the activation of natural killer cells and the release of inflammatory cytokines [23]. The adaptive immune response, which occurs several days later, includes the activation of T-cells and the production of specific antibodies against the measles virus. Studies have shown that measles infection can lead to long-term changes to the immune system, including increased susceptibility to other infections [22]. Lymphocytopenia and thrombocytopenia, often seen in measles, are a result of the immune-mediated destruction of these cells. The laboratory findings in this study, such as the median leukocyte count, haemoglobin concentration, platelet count and C-reactive protein, were all within normal ranges, similar to the median laboratory values reported in China. However, 14.5% (*n*=16), 59.1% (*n*=65), 0.1% (*n*=1) and 89.1% (*n*=98) of patients had leukopenia, anaemia, thrombocytopenia and raised C-reactive protein, respectively.

To the best of the authors’ knowledge, this is the first published study conducted in Somalia on measles. Overall, this study provides valuable insights into the clinical characteristics and outcomes of patients hospitalized with measles during an outbreak in Somalia. A strength of this study is the inclusion of a wide range of clinical and laboratory data. The study highlights the importance of timely vaccination and the need for improved care of patients with measles, particularly vulnerable groups including children and those with undernutrition. Public health interventions, such as increasing vaccination coverage and improving access to health care, are needed to reduce the burden of measles in Somalia.

However, this study also has some limitations. First, the retrospective design limits the ability to establish causality, in addition to underestimating deaths due to immigration. Secondly, there were only two deaths reported in this study, limiting further analysis into the factors associated with measles mortality in Somalia. Third, important variables, such as nutrition status, treatments given, and complications, were not captured in the patients’ files, making it impossible to infer associations and mortality seen in this study. Further research is needed to examine the low vaccine coverage rate and target interventions to improve this, and to assess risk factors for severe disease and death from measles, including underlying health conditions such as malnutrition and infections.

## Conclusions

Patients with measles in Somalia have a short hospital stay, low mortality rate and low vaccination rate. Timely vaccination and the need for improved care of patients with measles, particularly vulnerable groups including children and those with undernutrition, are encouraged.

## References

[1] World Health Organization (2014).

[2] (2013). Global Vaccine Action Plan. Decade of vaccine collaboration. Vaccine.

[3] Cockbain BC, Bharucha T, Irish D, Jacobs M. (2017). Measles in older children and adults. BMJ.

[4] United Nation's Children's Fund (2022).

[5] Rota PA, Moss WJ, Takeda M, de Swart RL, Thompson KM, Goodson JL. (2016). Measles. Nat Rev Dis Primers.

[6] Danova I. (2021). A review of measles virus. Prob Infect Parasit Dis.

[7] Stevens GA, Bennett JE, Hennocq Q, Lu Y, De-Regil LM, Rogers L (2015). Trends and mortality effects of vitamin A deficiency in children in 138 low-income and middle-income countries between 1991 and 2013: a pooled analysis of population-based surveys. Lancet Glob Health.

[8] Ahmed MA, Siewe Fodjo JN, Gele AA, Farah AA, Osman S, Guled IA (2020). COVID-19 in Somalia: adherence to preventive measures and evolution of the disease burden. Pathogens.

[9] Iacobucci G. (2022). Measles is now “an imminent threat” globally, WHO and CDC warn. BMJ.

[10] Dixon MG, Ferrari M, Antoni S, Li X, Portnoy A, Lambert B (2021). Progress toward regional measles elimination – worldwide, 2000–2020. MMWR Morb Mortal Wkly Rep.

[11] Portnoy A, Jit M, Ferrari M, Hanson M, Brenzel L, Verguet S. (2019). Estimates of case-fatality ratios of measles in low-income and middle-income countries: a systematic review and modelling analysis. Lancet Glob Health.

[12] Anis-ur-Rehman S, Idris M. (2008). Clinical outcome in measles patients hospitalized with complications. J Ayub Med Coll Abbottabad.

[13] Fetuga M, Njakanna O, Ongunfowora O. (2007). A ten-year study of measles admissions in a Nigerian teaching hospital. Niger J Clin Pract.

[14] Poletti P, Parlamento S, Fayyisaa T, Feyyiss R, Lusiani M, Tsegaye A (2018). The hidden burden of measles in Ethiopia: how distance to hospital shapes the disease mortality rate. BMC Med.

[15] United Nation's Children's Fund (2012).

[16] Martin-Canavate R, Custodio E, Yusuf A, Molla D, Fasbender D, Kayitakire F. (2020). Malnutrition and morbidity trends in Somalia between 2007 and 2016: results from 291 cross-sectional surveys. BMJ Open.

[17] Navarro-Colorado C, Mahamud A, Burton A, Haskew C, Maina GK, Wagacha JB (2014). Measles outbreak response among adolescent and adult Somali refugees displaced by famine in Kenya and Ethiopia, 2011. J Infect Dis.

[18] Mahamud A, Burton A, Hassan M, Ahmed JA, Wagacha JB, Spiegel P (2013). Risk factors for measles mortality among hospitalized Somali refugees displaced by famine, Kenya, 2011. Clin Infect Dis.

[19] World Health Organization. HIV: fact sheet on Sustainable Development Goals (SDGs): health targets. Copenhagen: World Health Organization Regional Office for Europe; 2017.

[20] United Nation's Children's Fund (2021). Measles vaccination coverage.

[21] Plans-Rubió P. (2020). Are the objectives proposed by the WHO for routine measles vaccination coverage and population measles immunity sufficient to achieve measles elimination from Europe?. Vaccines.

[22] Griffin DE. (2021). Measles immunity and immunosuppression. Curr Opin Virol.

[23] Griffin DE. (2016). The immune response in measles: virus control, clearance and protective immunity. Viruses.

